# Supplementary material for: Deletion of Transglutaminase 2 from Mouse Astrocytes Significantly Improves Their Ability to Promote Neurite Outgrowth on an Inhibitory Matrix
Source: Int J Mol Sci. 2023 Mar 23;24(7):6058. doi: 10.3390/ijms24076058 (PMC10094709; doi:10.3390/ijms24076058)
Supplement: Supplementary file 1 [file ijms-24-06058-s001.zip › ijms-2230537-supplementary.pdf]

Supplementary Material

Deletion of transglutaminase 2 from mouse astrocytes significantly improves their ability to promote neurite outgrowth on an inhibitory matrix

J. Emerson et al.

Figure S1

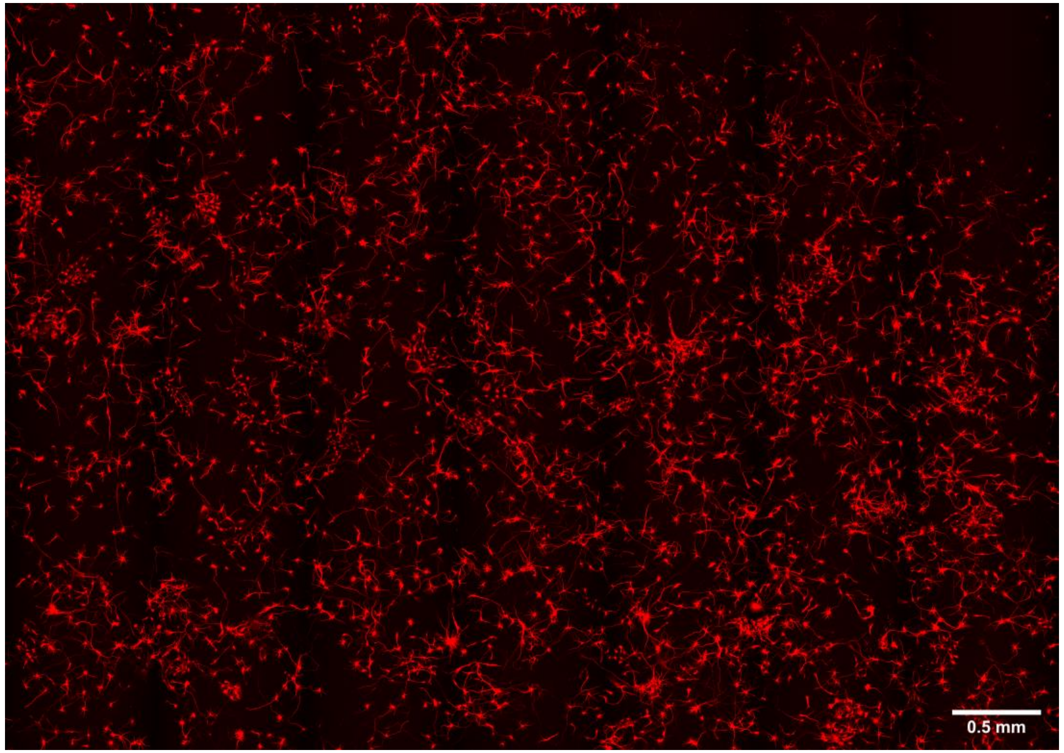

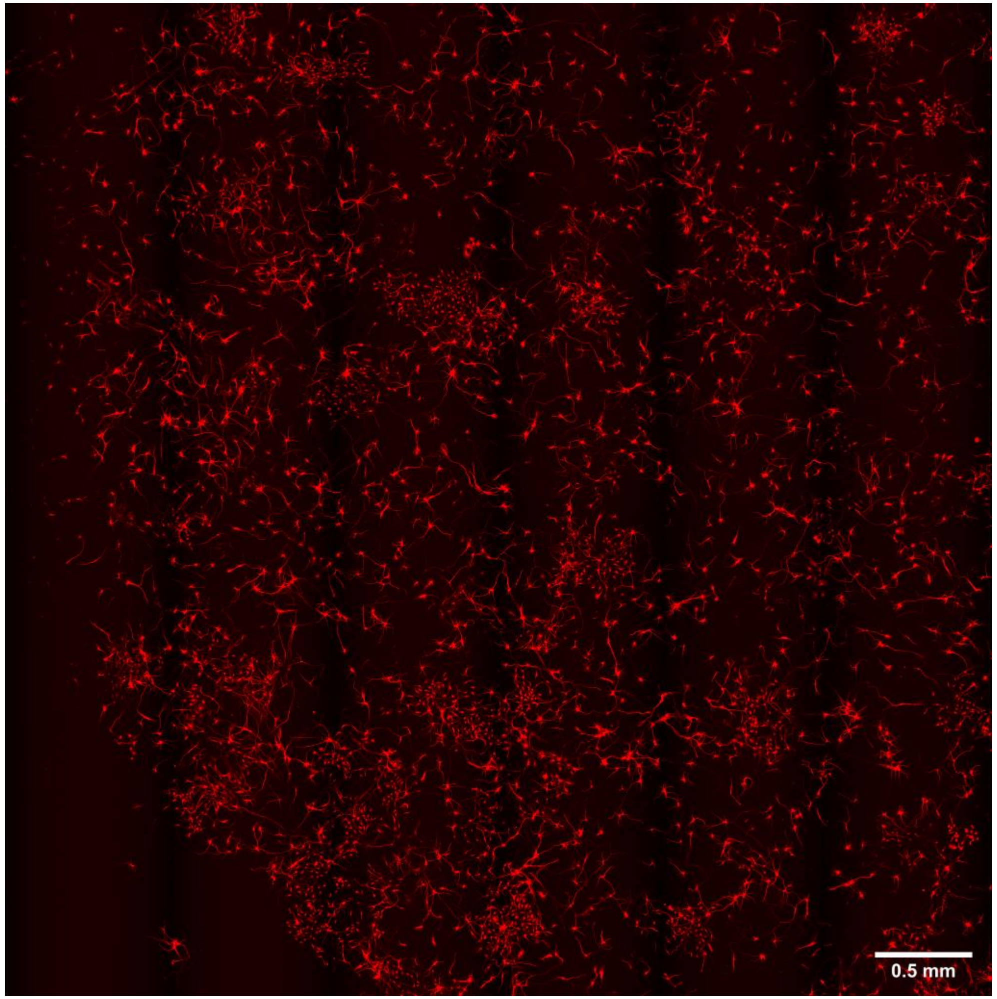

- Representative images of neurite outgrowth experiment showing MAP2 immunostaining of neurons on PDL from coverslips with high neuron-seeding density (24,000 cells/cm^2), approximately double the density used for the experiment in Figure 1. The edge of the coverslip is shown on the top right of the left image and bottom left of the right image. Neuron coverslips at DIV 5, that had the same initial seeding density, can slightly vary in their final cell density.

Figure S2

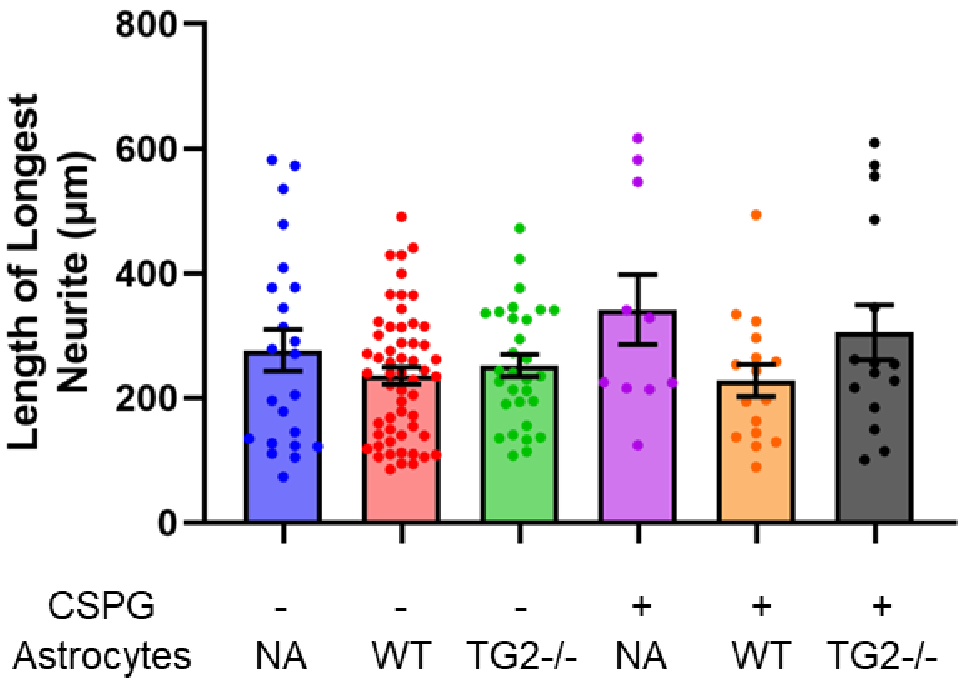

- Quantitation of neurite length on permissive and inhibitory matrices from coverslips with high neuron-seeding density show no significant differences between groups (24,000 cells/cm^2; n = 23-56 neurons per group on PDL and n = 10-16 neurons per group on CSPG).

Figure S3

tTG-Interacting Yeast Two-Hybrid Clones
Screened against: Human brain cDNA library
For each clone, 4 yeast strains were transformed according to the following pattern:
1) GAL4 binding domain (BD) in pGBD-C2 and GAL4 activation domain (AD) in pACT2
2) TG2-BD and pACT2
3) pGBD-C2 and Clone-AD
4) TG2-BD and Clone-AD

For each clone, each strain was grown to an OD600=0.1, and diluted 3x10 to make OD600=0.01, 0.001 and 0.0001; resulting colonies go from left to right on all images.
5 ul of each diluted clone was plated onto Complete Minimal (CM) Dropout Medium with glucose but lacking Leu and Trp (left) (control) or lacking Leu, Trp, His and Ade (right) (interaction). All clones should grow on CM Dropout Medium with glucose but lacking Leu and Trp (left) but only ones in which TG2-BD (bait) interacts with the AD-gene (prey from the library) will grow on the media lacking Leu, Trp, His and Ade (right panel, row 4).

SAP18 = sin3 associated polypeptide
GenBank: BC030836.1
Clone B338 (2004) aligns 155-909 – in frame

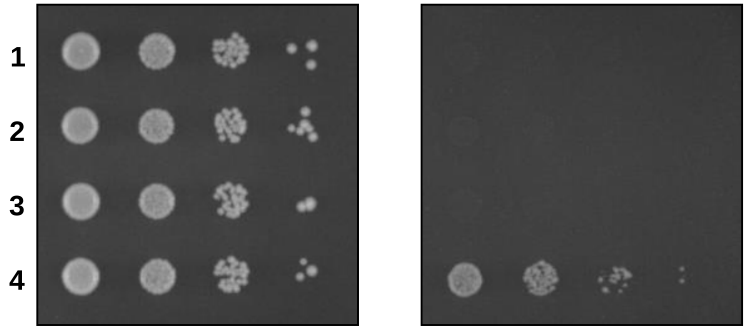

ZBTB7A = zinc finger and BTB domain-containing 7A
Genbank: NM_015898 CDS=760-1086
Clones 9 (2005); B4, B35, B119 and B187 (2004) align 387-1203 -Clones are in frame,

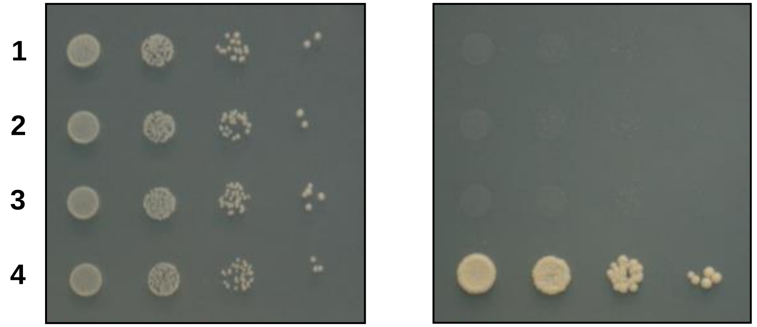

Figure S4

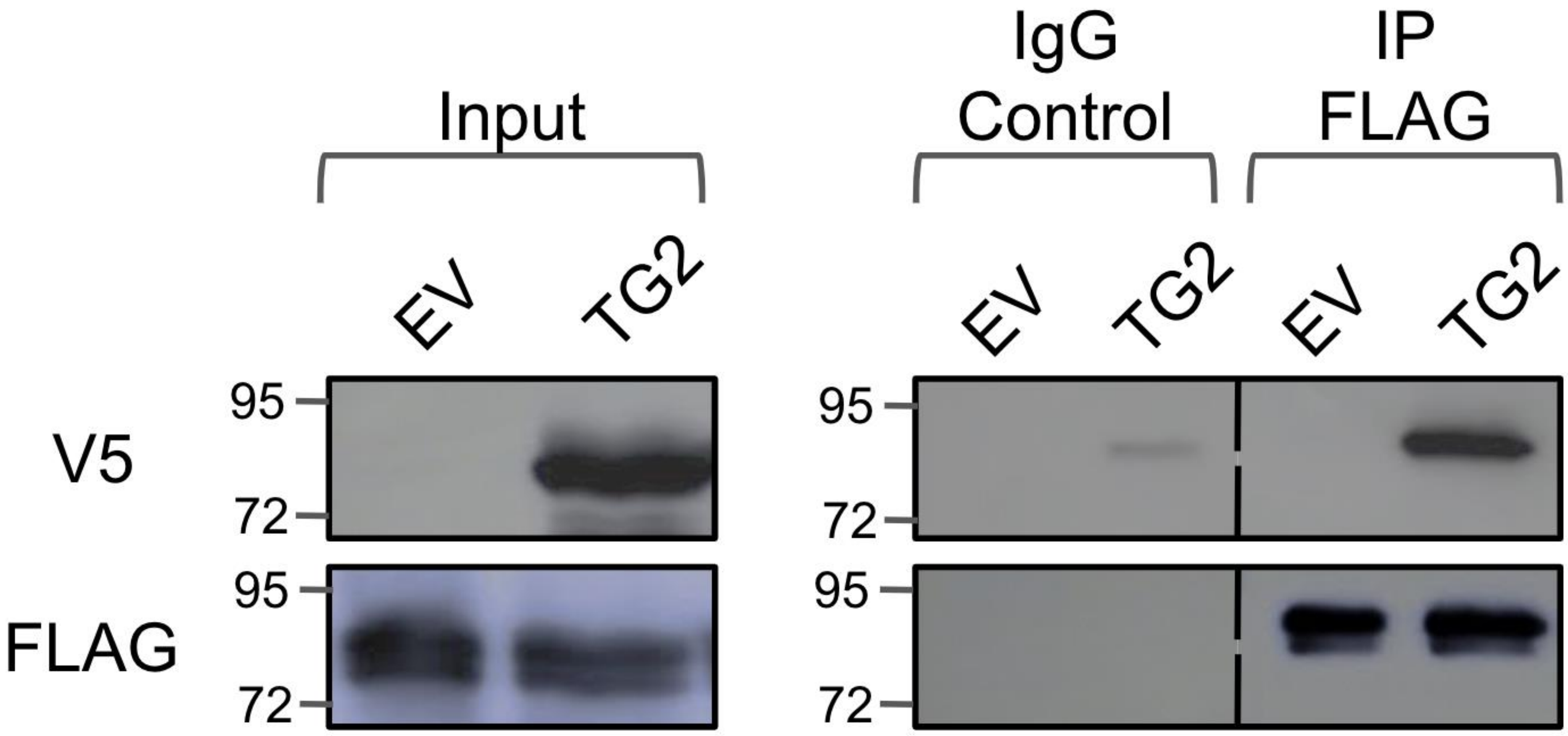

HEK cell IP of FLAG-Zbtb7a pull down V5-TG2

Figure S5

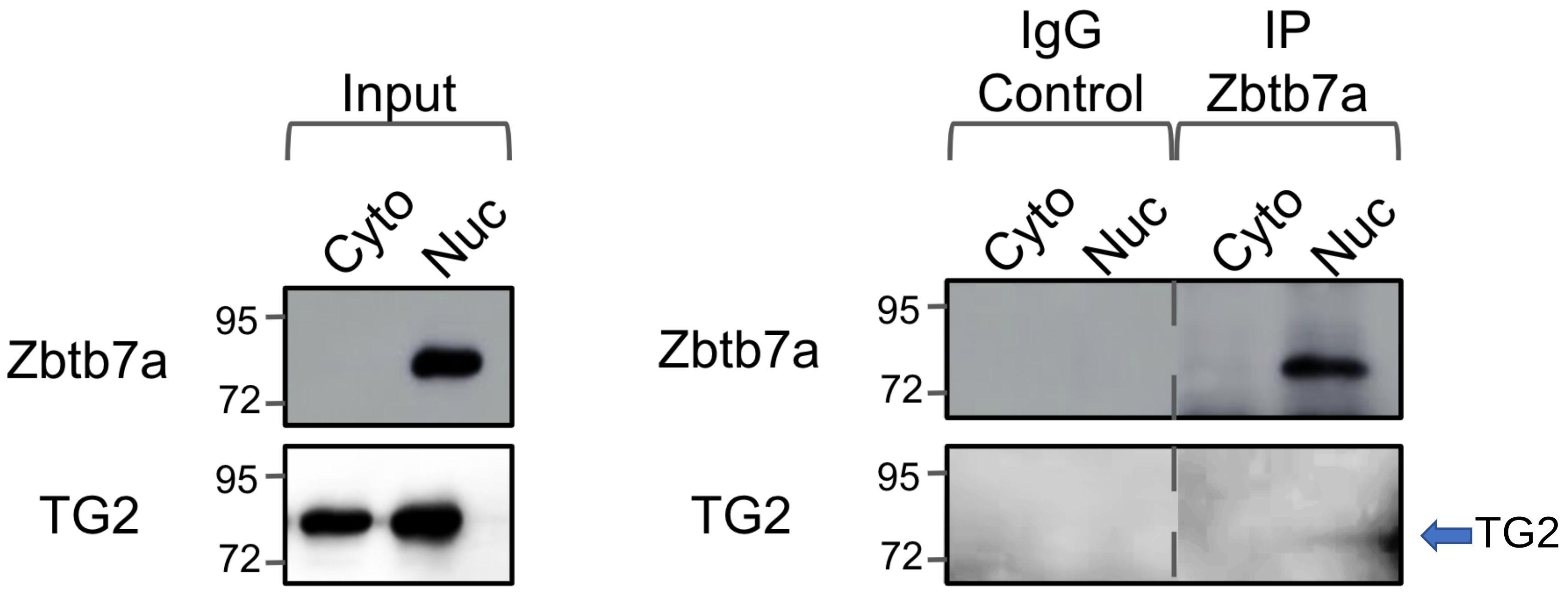

Astrocyte Endogenous IP of Zbtb7a pulls down TG2
